# Time- and Cost-Efficient Identification of T-DNA Insertion Sites through Targeted Genomic Sequencing

**DOI:** 10.1371/journal.pone.0070912

**Published:** 2013-08-12

**Authors:** Étienne Lepage, Éric Zampini, Brian Boyle, Normand Brisson

**Affiliations:** 1 Department of Biochemistry, Université de Montréal, Montréal, Quebec, Canada; 2 Institut de Biologie Intégrative et des Systèmes, Université Laval, Québec, Canada; Universidad Miguel Hernández de Elche, Spain

## Abstract

Forward genetic screens enable the unbiased identification of genes involved in biological processes. In Arabidopsis, several mutant collections are publicly available, which greatly facilitates such practice. Most of these collections were generated by agrotransformation of a T-DNA at random sites in the plant genome. However, precise mapping of T-DNA insertion sites in mutants isolated from such screens is a laborious and time-consuming task. Here we report a simple, low-cost and time efficient approach to precisely map T-DNA insertions simultaneously in many different mutants. By combining sequence capture, next-generation sequencing and 2D-PCR pooling, we developed a new method that allowed the rapid localization of T-DNA insertion sites in 55 out of 64 mutant plants isolated in a screen for gyrase inhibition hypersensitivity.

## Introduction

Genetic studies paved the way in understanding most biochemical processes in plants. Forward genetic screening requires mutant collections produced either by chemical mutagenesis using mutagens such as ethyl methanesulfonate, or by insertional mutagenesis, obtained by the agrotransformation of a T-DNA cassette [1]. The public availability of T-DNA insertion lines collections is particularly interesting as it considerably shortens the time required to perform a forward genetic screen. The function of many Arabidopsis genes have thus been characterized using the SALK collection, which is composed of more than 88,000 lines containing an inactivating T-DNA [2]. Additionally, collections of activation lines, transformed with a T-DNA containing repeated cauliflower mosaic virus (CaMV) 35 S enhancer regions, can also be used to characterize gene function [3], [4]. In these collections, in addition to disrupting the coding sequences, insertion of the T-DNA near the promoter region of a gene gives rise to overexpressors. These overexpressors allow the characterization of large families of genes, which might be redundant and generally missed by loss-of-function genetic screens [5].

The laborious work of mapping the T-DNA insertion sites in each mutant is one of the main problems that limit the use of large mutant collections for forward genetic screens. Many techniques have been proposed to identify T-DNA insertion sites, such as Tail-PCR [6], inverse PCR (IPCR) [7] and restriction site extension PCR (RSE-PCR) [8], but these methods present important limitations such as inefficient ligation step, the need of restriction enzymes that cut both the T-DNA and the genomic part at a reasonable distance and the generation of non-specific products through PCR [8]. Taken together, these limitations greatly affect the scalability and processivity of these techniques.

Recently, some studies have started to demonstrate the huge potential of next-generation sequencing to identify insertion sites. Indeed Illumina sequencing has been used to i) map the position of transposons in highly transposable maize lines [9], ii) identify insertion sites of LORE1 retrotransposon in *Lotus japonicus* [10], and iii) identify mutants for leaf shape abnormalities by the low-coverage sequencing of a pool containing genomic DNA from four distinct mutants [11].

Here, we describe targeted genomic sequencing, a new technique that allows the simultaneous identification of multiple insertion sites in a complex DNA sample. Using biotinylated primers specific for the extremities of the T-DNA, the regions flanking the insertion sites of 64 different mutants pooled together were specifically enriched from total genomic DNA. Roche GS-FLX + sequencing allowed the identification of 31 genes in which the T-DNA cassette was inserted. As an example, our approach is described with a screen carried out using gyrase inhibitors that specifically affect plant organelle genomes topology [12], [13], [14].

## Materials and Methods

### Plant Material, Growth Conditions

The Arabidopsis (*Arabidopsis thaliana*; ecotype Columbia-4) SK mutant collection was kindly provided by Dr. Isobel Parkin [3]. Seeds were sterilized, sown on Murashige and Skoog basal media (Sigma-Aldrich) supplemented with 1% sucrose and 0.8% agar and vernalised for 3 days in the dark at 4°C. Plants were grown under normal light (100 µmol m^−2^ s^−1^), at 22°C on a 16 h day/8 h dark cycle and the phenotype was assessed at seven days.

### Mutant Collection Screening

Approximately 150,000 plants of the SK collection were screened on 0.125 µM ciprofloxacin (CIP). Mutants presenting white sectors on the first true leaves were transplanted on soil to ensure their survival and allow them to set seeds. The progeny of each mutant was grown under the same conditions on medium containing either 0.125 µM CIP, 50 µM novobiocin (NOVO) or no drugs.

### Library Preparation

Total DNA was isolated for each plant sample using a cetyl trimethylammonium bromide (CTAB) DNA extraction protocol [15]. DNA from all samples was pooled in an equimolar ratio and a single GS-FLX+ rapid library was produced according to the manufacturer instructions (Roche, 454 Sequencing). The library was amplified by ligation mediated PCR using the 454 A and B primers as described in the general guidelines provided in the NimbleGen SeqCap EZ Library LR User's guide.

### Target Enrichment

Previous work demonstrated high specificity of 70 mer oligonucleotides in microarray analysis [16]. Therefore, three biotinylated 70 nucleotides long probes were designed to each extremity of the T-DNA sequence, ensuring that there was no significant sequence similarity to the Arabidopsis genome by blast analysis. The sequences are as follow: For the right border, RB1; AAC ATG GTG GAG CAC GAC ACT CTC GTC TAC TCC AAG AAT ATC AAA GAT ACA GTC TCA GAA GAC CAG AGG G, RB2; CTA TTG AGA CTT TTC AAC AAA GGG TAA TAT CGG GAA ACC TCC TCG GAT TCC ATT GCC CAG CTA TCT GTC A, RB3; TCA AAG ATA CAG TCT CAG AAG ACC AGA GGG CTA TTG AGA CTT TTC AAC AAA GGG TAA TAT CGG GAA ACC T. For the left border, LB1; ATG GAA ATT ATC TGC CTA ACC GGC TCA GTT CTG CGT AGA AAC CAA CAT GCA AGC TCC ACC GGG TGC AAA G, LB2; AAC GCC ATC CGA CGG ATG ATG TTT AAA AGT CCC ATG TGG ATC ACT CCG TTG CCC CGT CGC TCA CCG TGT T, LB3; AAG GTG CAC ATG GCT CAG TTC TCA ATG GAA ATT ATC TGC CTA ACC GGC TCA GTT CTG CGT AGA AAC CAA C. Target enrichment was performed using the SeqCap EZ hybridization and wash kit (Roche Nimblegen) using the general guidelines provided in the NimbleGen SeqCap EZ Library LR User's guide. Briefly, 10 µl of plant capture enhancer (Roche Nimblegen) and 5 µl of 100 µM hyb enhancing 454 A and B primers were added to 1 µg of amplified library and then dried. The A and B primers are added to inhibit unspecific interactions between the flanking primer regions in the DNA molecules of the library. The dried mixture was resuspended in 7.5 µl of 2X SC hybridization buffer and 3 µl of SC component A and heated to 70°C for 10 minutes. After a quick spin, 4.5 µl of the capture oligonucleotides solution in water (3.75×10^6^ molecules of each biotinylated oligonucleotide) were added. The amount of oligonucleotides added represents about a 5 fold excess of capture oligonucleotides to the number of genome molecules present. The hybridization mixture was incubated at 95°C for 10 minutes and then at 47.5°C for 40 hours. The hybridization mixture was put in contact with Streptavidin beads (Invitrogen) and non-captured material was washed away according to the NimbleGen SeqCap EZ Library LR User's guide. Captured material was amplified with the 454 A and B primers.

### GS-FLX + Sequencing and Analysis

Emulsion PCR and GS-FLX+ sequencing was performed according to manufacturer's instructions at the Plateforme d'Analyses Génomiques of the Institut de Biologie Intégrative et des Systèmes (Laval University, Québec, Canada). Raw sequencing reads were mapped to the T-DNA sequence vector (pSKI015– Genbank AF187951) and the Arabidopsis genome using the gsMapper module of Newbler v.2.5.3.We used the Linux/Unix grep command to rapidly pullout the lines containing the word “partial” from the 454ReadStatus.txt accessory file that is created by Newbler following the mapping of the reads to either the Arabidopsis genomes or the T-DNA vector. Alternatively, the 454ReadStatus file could have been filtered in a spreadsheet.

### 2D-PCR Pooling

Two distinct sets of 8 pools were prepared with an equivalent concentration of genomic DNA extracted from the different mutants in such a way that each mutant line is represented in a unique pool combination. PCR was conducted on 100 ng of the pooled genomic DNA with a T-DNA and a gene specific primer.

## Results

### Chemical Screening using Gyrase Inhibitors Identified 64 Sensitive Plants

**Figure 1:**
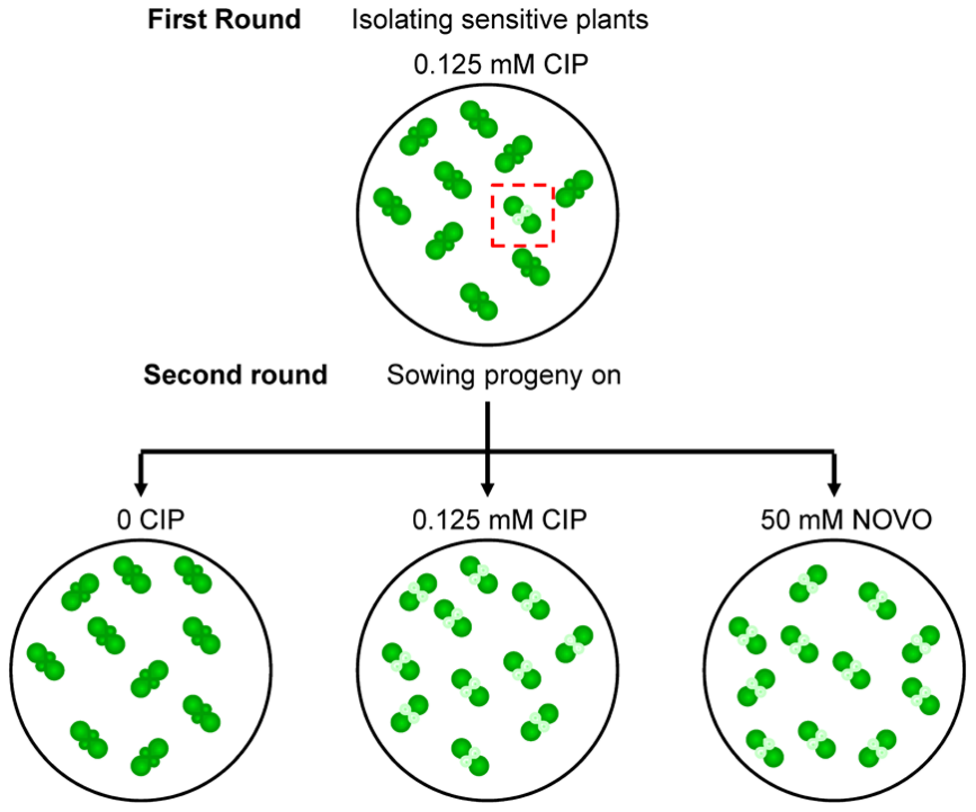
Forward Genetic Screen to Identify Genes Involved in the Maintenance of Organelle Genome Topology. Schematic representation of the different steps of the forward genetic screen. Plants with white first true leaves represent the mutants sensitive to ciprofloxacin (CIP) or novobiocin (NOVO).

To identify the genes involved in the maintenance of organelle genome topology, we set up a screen for gyrase inhibition hypersensitivity using the SK collection, which is composed of approximately 55,000 different activation tagged lines [3]. A global view of the screen is summarized in Figure 1. The first round of screening consisted of growing 150,000 tagged lines on 0.125 µM ciprofloxacin (CIP), a gyrase inhibitor that introduces DNA double strand breaks (DSBs) in plant organelle genomes, and isolating the plants that showed signs of hypersensitivity [17]. Progeny of these plants were then submitted to a second round of screening, in the presence or absence of CIP (Figure 1). In addition, plants were also germinated on a medium containing novobiocin, a gyrase inhibitor that does not introduce DSBs [18]. The second round of screening identified 64 plants hypersensitive to CIP, of which eight were sensitive to both novobiocin and ciprofloxacin. We then proceeded to identify the T-DNA insertion sites for all the 64 plants.

### Targeted Genomic Sequencing for High-Throughput Insertion Sites Identification

**Figure 2:**
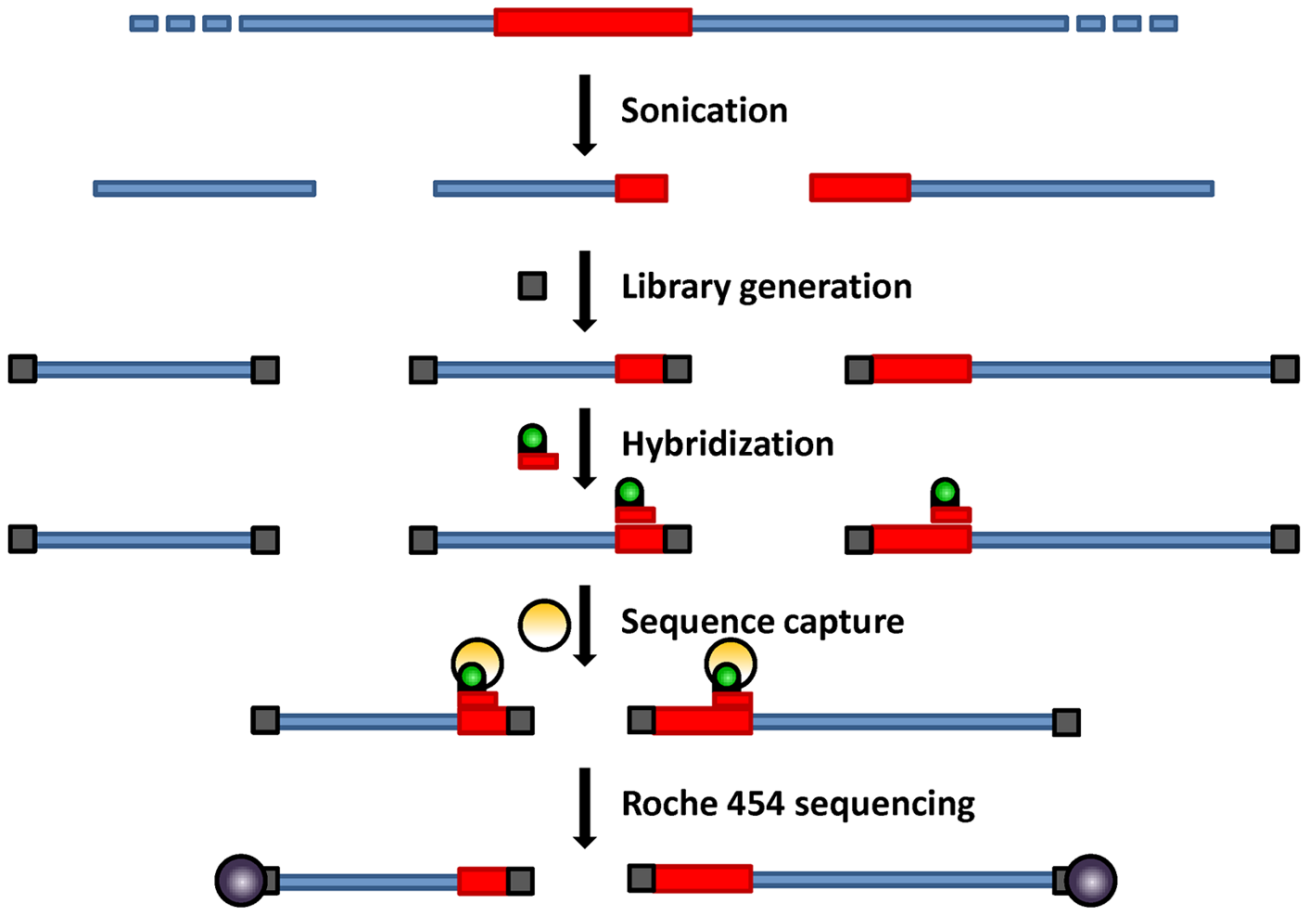
Overview of Targeted Genomic Sequencing. Blue rectangles represent genomic DNA, and red rectangles correspond to T-DNA insertions. The grey squares represent the 454 specific primers added in order to bind the sequencing beads (purple circles). The green circles correspond to biotin bound to a red T-DNA specific primer and hybridized to T-DNA. Hybridized sequences are then enriched by capture on streptavidin beads (orange circles).

**Figure 3:**
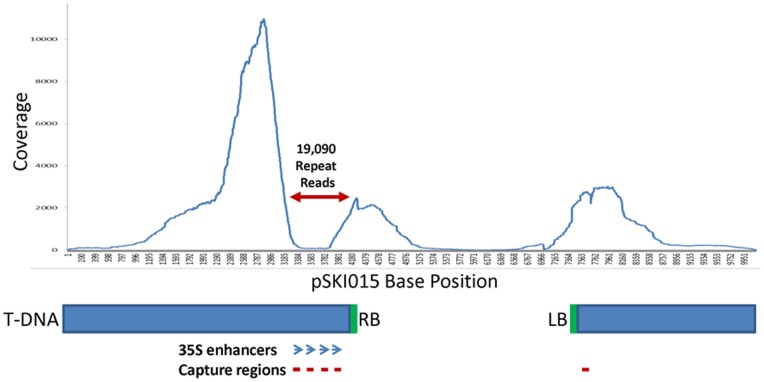
Coverage of the pSKI015 Vector Obtained by Sequencing. Features of the pSKI015 are summarized below the coverage graph. The blue rectangles represent the T-DNA cassette with the right (RB) and left (LB) borders in green. The position of the 35 S enhancers are indicated by blue open end arrows. The red lines represent the annealing regions of the three biotinylated primers for each border. The position where the repeated reads align is indicated by the double red arrowhead line on the coverage graph.

The identification of T-DNA insertion sites in 64 plants by classical methods such as TAIL-PCR or IPCR represents a highly laborious and time-consuming task [8]. However, given the recent advances in next-generation sequencing coupled with targeted gene enrichment, it was foreseeable to achieve multiple insertion sites identification in a cost and time efficient manner. Figure 2 describes the different steps used in this method. Briefly, a next-generation shotgun library was prepared with a pool containing equivalent amounts of CTAB-extracted genomic DNA from every line. Then, biotinylated primers complementary to the T-DNA ends were hybridized to the genomic DNA library and hybridized target T-DNA recovered using the SeqCap EZ hybridization kit. It was expected that the regions flanking the T-DNA insertion sites would be enriched as well during this procedure. Following amplification of the captured material using 454 specific primers, the efficiency of the T-DNA capture was assessed by qPCR. Finally, ROCHE 454 GS-FLX + sequencing was carried out to identify the region flanking the T-DNA in each line. A major advantage of GS-FLX + is that the read length can reach a thousand bases, increasing the probability of getting a hybrid fragment composed of a T-DNA and a genomic part. The sequencing reads were aligned against the T-DNA sequence using the gsMapper module of Newbler v2.5.3. Of the 115,000 reads obtained, 28,023 reads mapped to a unique position of the T-DNA cassette and 19,090 reads mapped to the enhancer repeat region, indicating that the sequence capture worked efficiently, with more than 40% of the reads mapping to the T-DNA (Figure 3). Most of these reads fully mapped to the T-DNA. Reads mapping closely to the T-DNA right border were more abundant compared to the ones mapping closely to the left border, most likely due to the design of the probes within the 4x repeated CaMV 35S enhancer adjacent to the right border (Figure 3). Nevertheless, approximately 4,000 reads that partially mapped to the T-DNA had a remaining portion also mapping to the Arabidopsis genome. The sequencing identified 31 genes, suggesting that more than one plant could be mutated for the same gene.

### Tracking the Mutations by Pool-PCR

**Figure 4:**
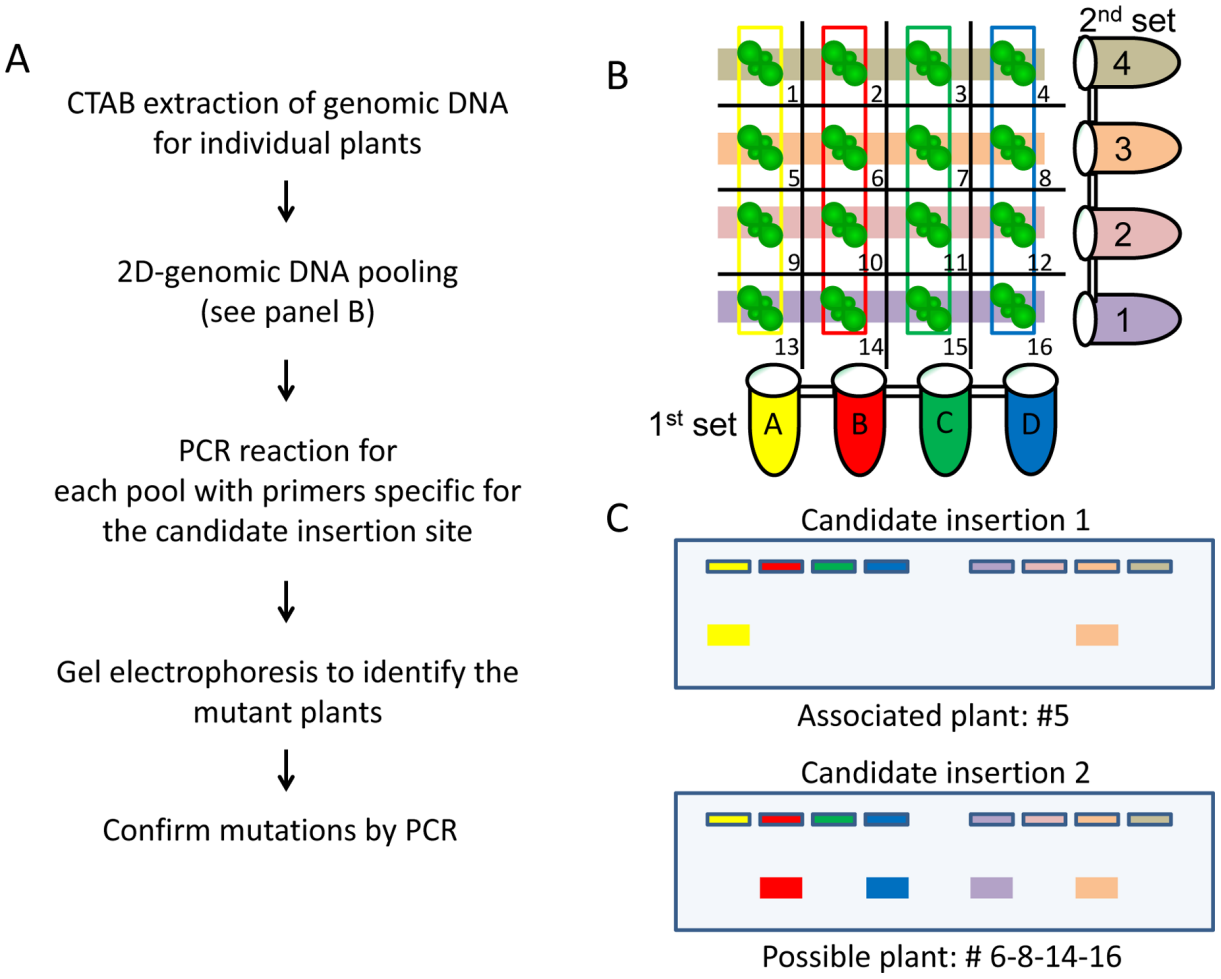
Association of an Insertion Event to a Specific Line by 2D-PCR Pooling. A. Workflow of the 2D-PCR pooling B. An example of the pooling design for 16 plants. Each plant genomic DNA is pooled in a unique set combination. The plants encompass by the colored rectangle associate to the pool of the same color. C. Data analysis to identify the positive line. All bands on a given gel correspond to the same amplification product in different pools.

Many next-generation sequencing approaches take advantage of barcoding, which consists of adding a unique short DNA sequence to each genomic sample to easily distinguish individuals. However, library production costs can be prohibitive when dealing with many different samples. In order to minimize these costs, 2D-PCR pooling was used in the present study to match the identified mutations to each CIP-sensitive plants (Figure 4). Genomic DNA from the 64 CIP-sensitive plants was split into two sets of eight pools in a manner ensuring that each genomic sample would be present in a unique set combination [19]. Figure 4B presents an example for the preparation of the pools for 16 plants. Then a specific PCR reaction was carried out for every candidate gene (31 in our case) and the amplified products were separated on gel. Depending on the number of lines that carries the tested insertion, a specific pattern of band is obtained. When the mutation is present only once, a single band is detected in the 1^st^ and 2^nd^ pool which can be linked to a single plant (Figure 4C, upper part). When more than one plant has the insertion, different outcomes are possible and require confirmation of the insertion for every candidate gene (Figure 4C, lower part). However, even when dealing with the first scenario, it is always best to confirm the insertion with a second PCR.

**Figure 5:**
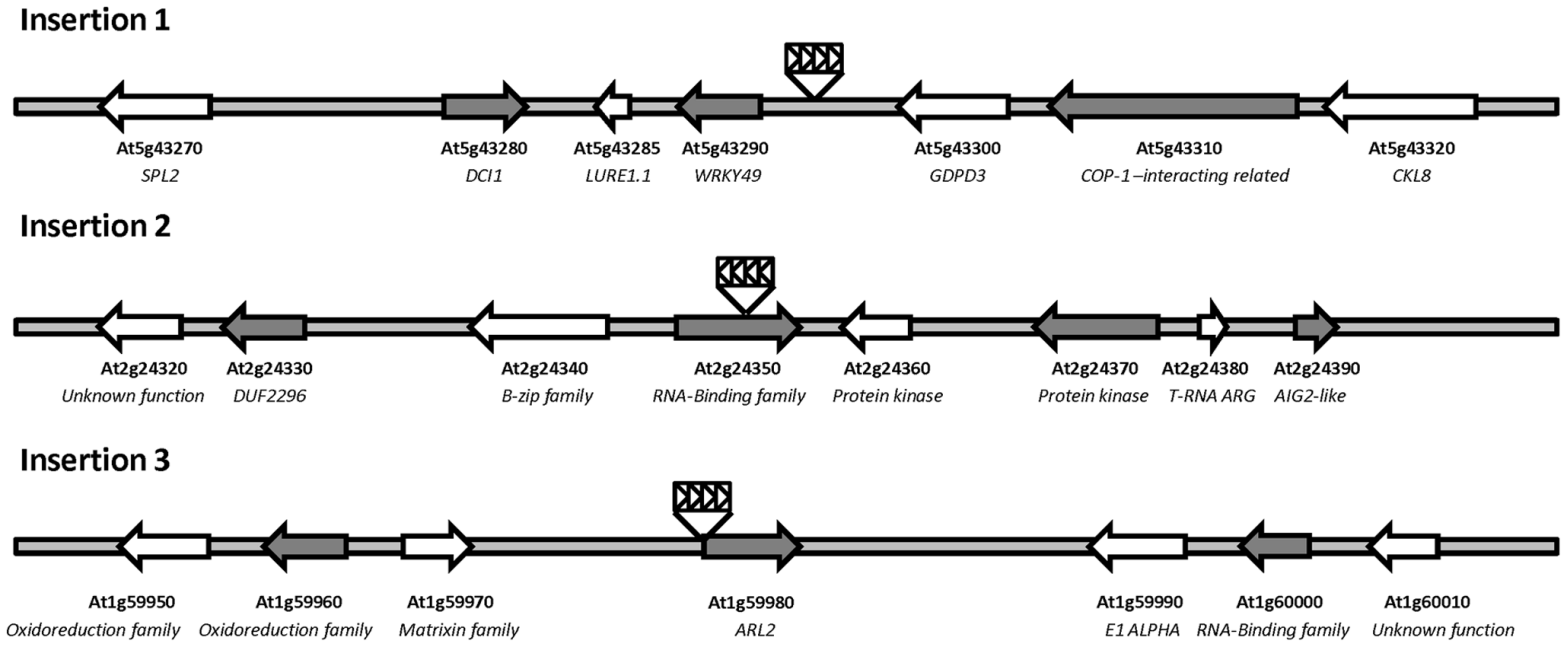
Schematic Illustration of the Insertion Sites in the Three Novobiocin-Sensitive Mutant Lines. The small black arrows represent the orientation of the CaMV 35S enhancers within the T-DNA (rectangle). For Insertion 3, a different part of the plasmid still containing the enhancer region has been inserted.

Although more time-consuming than barcoding, this straight-forward approach was shown to be very efficient as 86% of the plants (55/64) could be successfully genotyped. The remaining nine plants either contain an insertion site that could not be identified by our technique or other DNA modifications that would cause the phenotype. From the 64 plants isolated by the screen, eight were hypersentive to both ciprofloxacin and novobiocin, which suggests a role for the mutated genes in the control of organelle genome topology, and 56 plants were solely sensitive to ciprofloxacin. By 2D-PCR pooling, we determined that the eight mutants sensitive to both gyrase inhibitors were only associated to three different insertions and thus represent three lines. Two plants were mutated in the 5′ UTR of the *ARL2* gene (AT1G59980), three in the intergenic space between *Wrky49* (AT5G43290) and *GDPD3* (AT5G43300), and three in the 6^th^ intron of AT2G24350. It is not surprising that we obtained more than one plant for these lines as we screened more plants than the total expected number of lines present in the collection. The mapping of the insertion sites of these three different lines is presented in Figure 5. The other 28 mutations, which we could associate to 47 of the 64 isolated plants, were not found among the eight CIP/NOVO-sensitive plants and will be described elsewhere.

## Discussion

In this report we describe a time-efficient and low cost targeted genomic sequencing technique for high-throughput identification of insertion lines in forward genetic screens. Indeed, with the combination of next-generation sequencing and 2D-PCR pooling, we show that it is possible to identify most insertion sites in a large collection of mutants. The approach described here to capture the T-DNA ends is very efficient since more than 40% of the reads obtained corresponded to the T-DNA cassette. This is particularly impressive considering the complexity of the genomic sample used, which was composed of a genomic DNA pool from 64 different genomic extractions. The fact that a majority of the captured sequences corresponded to the T-DNA right border, which contains the repeated CaMV 35 S enhancer, indicates that repetitive sequences should be avoided from primer design since it leads to over representation of this sequence, therefore increasing background noise.

The efficiency of the technique was demonstrated in a forward genetic screen for gyrase inhibition hypersensitivity. In this screen, we isolated 56 plants sensitive specifically to ciprofloxacin and 8 sensitive to both ciprofloxacin and novobiocin. The mutants sensitive specifically to ciprofloxacin might be involve in the maintenance of organelle genome stability, since ciprofloxacin generates DNA double-strand breaks (DSBs) in the organelles. Conversely, novobiocin inhibits organelle gyrases without inducing DSBs, thus the phenotype observed for the 3 lines (8 isolated plants) also sensitive to novobiocin would most likely be related to gyrase inhibition itself. Given the depth of the sequencing, with more than 4000 reads representing the 31 genes identified by the screen, it seems likely that no other insertional mutation would be present in those three lines. Nevertheless, we cannot exclude that the phenotype observed in those lines could be linked to an insertion that could not be identified by our technique or a DNA rearrangement induced by the agrotransformation process [20].

The approach reported here to identify T-DNA insertion sites on a large scale presents several advantages over other NGS techniques. Because of the sequence capture step that eliminates most unwanted genomic DNA, it is more scalable than the low coverage sequencing approach described by Polko et al. (2012), which is one of the least expensive NGS techniques available. In addition, the 2D-PCR pooling step significantly reduces the cost of our approach as compared to the Illumina barcoded library preparation [9]. Finally, the use of Roche 454 GS-FLX + facilitates the identification of flanking sequences when dealing with variable borders, such as those present in T-DNA cassettes, by giving longer reads and clear hybrid T-DNA/ flanking region fragments. On the other hand, one must consider that 2D-PCR pooling is more time-consuming than barcoding, as separate PCR reactions must be carried out to confirm each insertion. Altogether, our technique is well suited to identify mutants isolated from a forward genetic screen that has a limited number of insertions (usually 1 to 5), at a medium throughput. However 2D-PCR becomes too laborious when dealing with numerous insertions, which is the case in most retrotransposon experiments, or in very large mutant collections. Although 3D-PCR pooling [19] and more in depth sequencing could provide a higher throughput, this would still be more laborious than barcoding.

A great advantage of our procedure is that a single 454 library is made from multiple samples, followed by a single capture experiment requiring a single sequencing run that leads to the identification of more than 80% of the insertions sites. The whole process from DNA to sequencing reads takes only 2 weeks. In comparison, classical PCR based techniques needs to be performed on individual samples and requires 2 to 3 nested PCR steps that often lead to multiple PCR bands. This is in addition of the important limitations such as the inefficient ligation step or the need for restriction enzymes that cut both the T-DNA and the genomic part [8]. A hybrid version of classical and NGS approaches has recently been described in *Lotus japonicus* [10]. PCR amplification of flanking regions of a retrotransposon was used to enrich for the junctions between retrotransponsons and genomic DNA prior to Illumina sequencing on a single barcoded pool. The PCR amplification efficiency was superior to our sequence capture approach yielding 73% of the reads linked to the retrotransposon insertion sites. This technique has the advantage of being more scalable while still being affordable. However, the variable length and structure of T-DNA borders inserted in Arabidopsis [3] would greatly complicate the design of primers and be a hindrance for the PCR amplification required in this technique. This problem is solved by the sequence capture step described here. The simplicity and rapidity of our procedure compared to PCR-based methods are its most attractive features.

In conclusion, we show that the efficiency of sequence capture and 2D-PCR pooling can greatly diminish the cost of next-generation sequencing when dealing with many different biological samples. Furthermore, this technique is not restricted to the T-DNA sequence or to Arabidopsis, but is also suitable for the identification of unknown regions flanking any known DNA sequence, in any organism.
